# Consumption of trematode parasite infectious stages: from conceptual synthesis to future research agenda

**DOI:** 10.1017/S0022149X23000111

**Published:** 2023-03-27

**Authors:** J. Koprivnikar, D.W. Thieltges, P.T.J. Johnson

**Affiliations:** 1 Department of Chemistry and Biology, Toronto Metropolitan University, 350 Victoria Street, Toronto, ON, Canada M5B 2K3;; 2 Department of Coastal Systems, NIOZ Royal Netherlands Institute for Sea Research, Den Burg, The Netherlands; 3 Department of Ecology and Evolutionary Biology, University of Colorado, Boulder, CO, USA

**Keywords:** Trematode, helminth, cercaria, consumption, food web, trophic, ingestion

## Abstract

Given their sheer cumulative biomass and ubiquitous presence, parasites are increasingly recognized as essential components of most food webs. Beyond their influence as consumers of host tissue, many parasites also have free-living infectious stages that may be ingested by non-host organisms, with implications for energy and nutrient transfer, as well as for pathogen transmission and infectious disease dynamics. This has been particularly well-documented for the cercaria free-living stage of digenean trematode parasites within the Phylum Platyhelminthes. Here, we aim to synthesize the current state of knowledge regarding cercariae consumption by examining: (a) approaches for studying cercariae consumption; (b) the range of consumers and trematode prey documented thus far; (c) factors influencing the likelihood of cercariae consumption; (d) consequences of cercariae consumption for individual predators (e.g. their viability as a food source); and (e) implications of cercariae consumption for entire communities and ecosystems (e.g. transmission, nutrient cycling and influences on other prey). We detected 121 unique consumer-by-cercaria combinations that spanned 60 species of consumer and 35 trematode species. Meaningful reductions in transmission were seen for 31 of 36 combinations that considered this; however, separate studies with the same cercaria and consumer sometimes showed different results. Along with addressing knowledge gaps and suggesting future research directions, we highlight how the conceptual and empirical approaches discussed here for consumption of cercariae are relevant for the infectious stages of other parasites and pathogens, illustrating the use of cercariae as a model system to help advance our knowledge regarding the general importance of parasite consumption.

## Introduction

Over the past three decades, scientists have increasingly recognized the need to integrate parasites into food webs and characterize their effects on such topologies (Marcogliese & Cone, 1997; Lafferty *et al*., 2006a, 2008; Dunne *et al*., 2013). This is especially true given that many parasites do not just affect trophic interactions, but can also be consumed. There are two main routes by which such consumption may occur (Lafferty *et al*., 2008; Johnson *et al*., 2010; Lopez & Duffy, 2021). In one route, predators can consume infected hosts containing adult or larval parasites (concomitant predation). This can lead to trophic transmission if the predator is a suitable next host for parasite, or to parasite death or expulsion if not.

For the second route, free-living infectious or resting stages – which are used by many parasites and pathogens to spread among hosts – can be consumed by non-host organisms (Johnson *et al*., 2010). Given that free-living parasite and pathogen stages are ubiquitous and can represent tremendous biomass in some systems, studies have increasingly paid attention to their potential consumption. For instance, marine viruses are not only the most numerically abundant biological entities within oceans, but are consumed by many marine organisms (Welsh *et al*., 2020). Consumption of infectious stages has been reported from a range of different systems and taxa, from aquatic to terrestrial and from microparasites to macroparasites (see reviews by Johnson & Thieltges, 2010; Johnson *et al*., 2010). As further examples, various freshwater zooplankton consume juvenile nematodes (Achinelly *et al*., 2003) and the fungal zoospores that cause chytridiomycosis in amphibians (Searle *et al*., 2013), while numerous free-living amoebae predate upon pathogenic bacteria (Bornier *et al*., 2021). By reducing encounter with potential hosts, the consumption of free-living parasite stages also has broad implications for infectious disease dynamics, from modifications in transmission to intensity-dependent pathology (Johnson *et al*., 2010; Lopez & Duffy, 2021; Shaw & Civitello, 2021).

Free-living infectious stages are particularly central to the complex life cycles of many parasitic helminths (Parker *et al*., 2003). We thus contend that accounting for the consumption of free-living helminth stages is vital to understanding how parasites influence food web structure, with more and more studies noting the importance of including larval parasites as prey (e.g. Lafferty *et al*., 2006b; Cirtwill & Stouffer, 2015; Michalska-Smith *et al*., 2018). The roles of helminths in food webs can thus range from that of a free-living basal resource on one extreme if we consider that their non-feeding infectious stages can be consumed by a variety of organisms, to that of a top consumer such as a predator on the other extreme by feeding on their hosts (Cirtwill & Stouffer, 2015; Sures *et al*., 2017).

Food webs that omit parasites in their better-known roles as consumers already overlook ~50% of trophic links (Dunne *et al*., 2013), let alone accounting for their more cryptic roles (e.g. as a basal resource). However, most of the food webs that have incorporated parasite consumption did so with a focus on concomitant predation (e.g. Dunne *et al*., 2013; Poulin *et al*., 2013; Cirtwill & Stouffer, 2015; Michalska-Smith *et al*., 2018; Morton *et al*., 2021) and few to date have considered predation upon free-living stages (e.g. Thompson *et al*., 2005; Lafferty *et al*., 2008). There is therefore a need to more explicitly consider the latter, and in the few cases where such direct consumption has been examined, it accounts for the same proportion of links as trophic transmission (Thieltges *et al*., 2013).

**Fig. 1.**
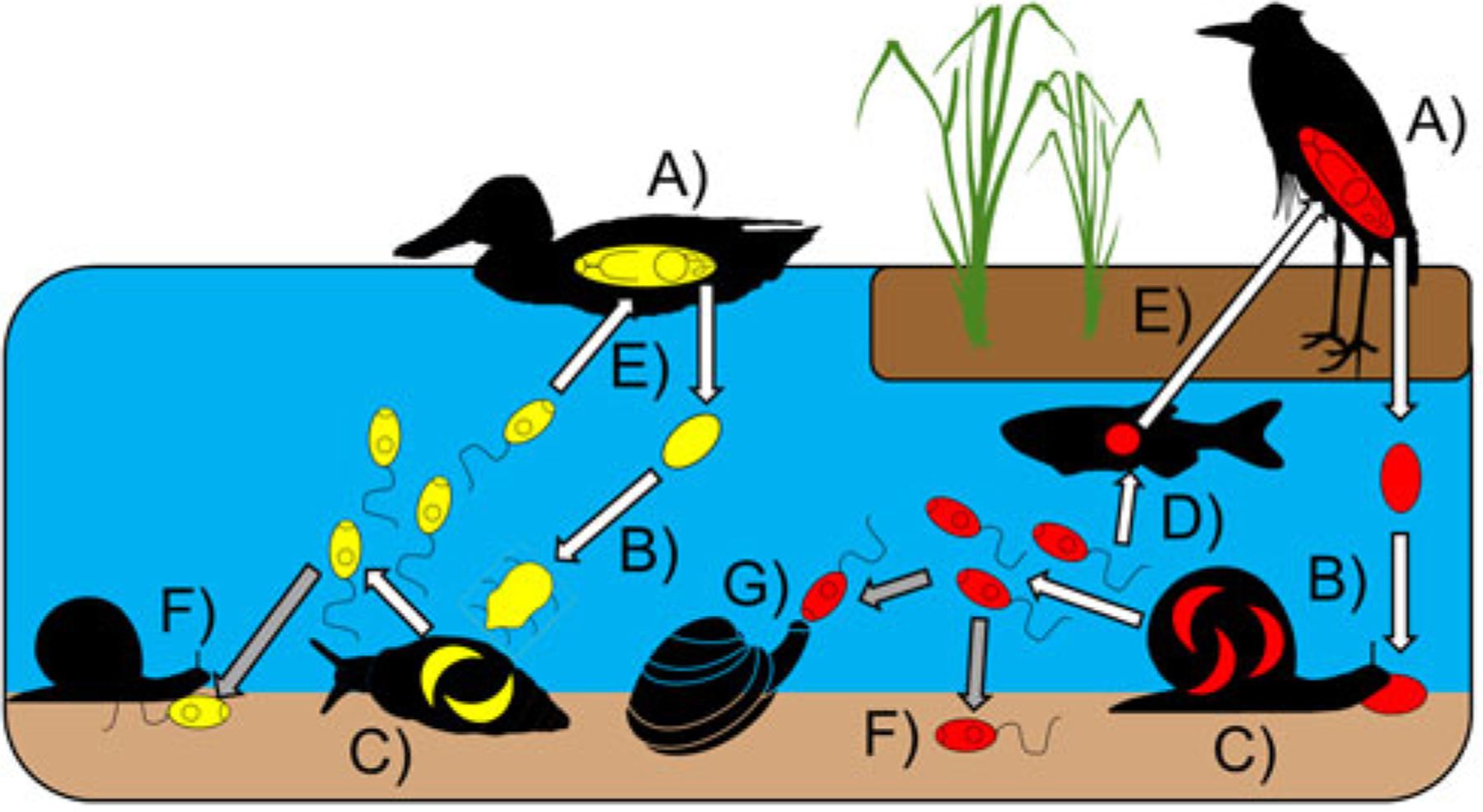
Basic trematode life cycles (white arrows) of two species (red and yellow) and their transmission pathways (grey arrows). (a) Adult worms in definitive/final host sexually reproduce and release eggs into environment; (b) first intermediate host becomes infected by consuming eggs or by a penetrating miracidium hatched from an egg; (c) asexual reproduction by parthenitae (sporocysts or rediae) generates cercariae that emerge into environment; (d) cercaria forms a cyst (metacercaria) in second intermediate host; (e) life cycle complete through final host consumption of infected second intermediate host or via direct penetration of cercariae; (f) inactive and dead cercariae sink to benthos and may be consumed; (g) live cercariae may be consumed.

Here, we focus on the consumption of trematode cercariae, a primarily aquatic free-living infectious stage of helminths within the Class Trematoda (Phylum Platyhelminthes) – see fig. 1 and below for general life cycle description. Not only are trematodes abundant and diverse in aquatic systems, from intertidal to freshwater (Kuris *et al*., 2008; Preston *et al*., 2013), they are often also the most prominent macroparasites in food webs (e.g. Morton *et al*., 2021). Of particular note is the substantial biomass reported for cercariae (Kuris *et al*., 2008; Preston *et al*., 2013, 2021; Soldánová *et al*., 2016; Rosenkranz *et al*., 2018), often collectively exceeding that of free-living taxa such as birds or aquatic insects. Consequently, cercariae represent a readily-available prey item in many habitats (Johnson & Thieltges, 2010; Johnson *et al*., 2010) and their consumption is well-studied relative to that of other parasite infectious stages given the number of trematode species with medical, economic and wildlife importance (Toledo & Fried, 2019). Parasites and pathogens may thus directly contribute to secondary production by serving as a food resource (Preston *et al*., 2016; Fischhoff *et al*., 2020), especially through the consumption of free-living infectious stages that are abundant in the environment, such as cercariae.

Beyond documenting the types of parasite free-living infectious stages that can be consumed by various predators, a range of studies have also noted the potential epidemiological implications of such parasite removal. These primarily relate to reduced parasite transmission to hosts downstream in the life cycle (Thieltges *et al*., 2008a; Johnson *et al*., 2010), and it has been suggested that predation pressure might be substantial for most parasite life history stages (Thieltges *et al*., 2013). Such consumption of parasites present within the environment extends the original concept proposed for the ‘dilution effect’ of biodiversity (Keesing *et al*., 2006) by expanding it to include non-host organisms when they remove free-living infectious stages (Johnson & Thieltges, 2010; Johnson *et al*., 2010), thereby reducing encounter rates between parasites and competent hosts.

Given the substantial number of studies (table 1) that have reported consumption of cercariae (also known as cercariae consumption), along with increasing recognition that parasites have substantial roles and influences within food webs (Thompson *et al*., 2005; Lafferty *et al*., 2008; Dunne *et al*., 2013), it is timely to take a broader ecological and epidemiological perspective by synthesizing work to date. For instance, are there particular, universal features of cercariae across trematode species and habitats that make them more vulnerable to predation? Are there certain consumer traits that increase the likelihood of ingesting cercariae? Is cercariae consumption high enough to affect either parasite transmission or consumer growth/population dynamics? This will also aid in generalizations regarding the consumption of free-living parasite and pathogen infectious stages as a whole, as well as highlighting gaps in knowledge and key avenues for future research. The body of studies regarding transmission-related effects of cercariae consumption also provide similar opportunities to identify broad patterns and opportunities for further work. For instance, are consumers in particular feeding guilds more effective at reducing transmission of cercariae to downstream hosts?

We aim to highlight the following in this review and synthesis: (a) approaches in studying cercariae consumption; (b) the range of consumers and trematode prey documented thus far; (c) factors influencing cercariae consumption; (d) individual-level consequences of cercariae consumption (e.g. their viability as a food source); (e) community-level and ecosystem-level implications of cercariae consumption (e.g. transmission, nutrient cycling and influences on other prey); and (f) gaps in our knowledge and future directions – specifically, our ability to predict when cercariae–consumer interactions are likely to be strong, as well as speak to the magnitude and nature of parasite consumption effects in natural ecosystems. As the conceptual and empirical approaches and findings for cercariae in this context are likely relevant for the infectious stages of other parasites and pathogens, focusing on cercariae as a model system will help to advance our knowledge regarding the importance of parasite consumption.

## Trematode cercariae as a system for understanding parasite consumption

### Free-living stages in trematode life cycles

Most trematodes within the subclass Digenea have a three-host life cycle (see fig. 1), with adult worms residing and sexually reproducing within the digestive tracts of vertebrate definitive hosts who then expel parasite eggs into the environment along with their own waste (Esch *et al*., 2002). The required molluscan first intermediate hosts (typically gastropods) become infected by ingesting eggs containing infective miracidia, or by miracidia that hatch and actively seek them out. Once in the molluscan digestive–gonadal complex, miracidia ultimately develop into asexually-reproducing stages collectively known as parthenitae that derive their nutrients and energy from their host. Cercariae produced within these parthenitae (sporocysts or rediae) emerge from their molluscan host into the environment. Cercariae are non-feeding, relying on energy stores, and typically live about 24 h while seeking out their next host (e.g. Evans & Gordon, 1983), with lifespans showing variation among species and environmental conditions, as well as whether they use two or more hosts to complete their life cycle (fig. 1).

**Fig. 2.**
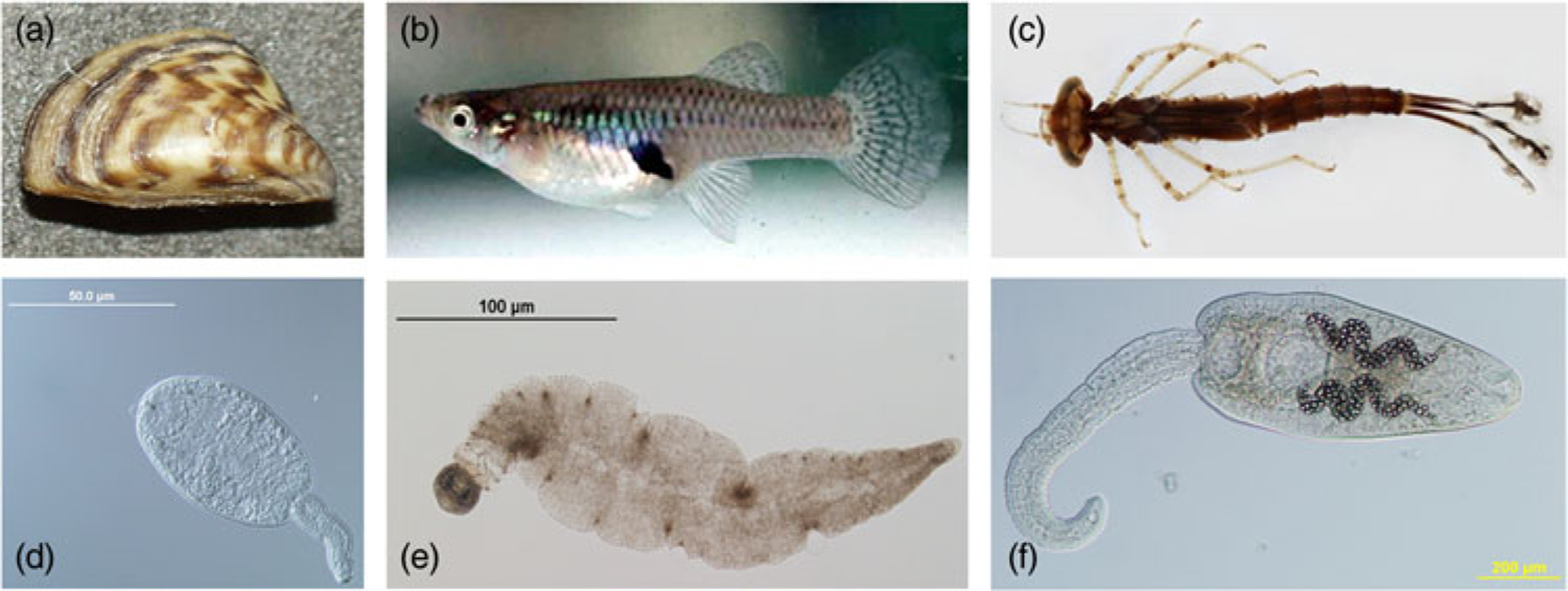
Select examples of different consumers of trematode cercariae. (a) Filter-feeder (zebra mussel, https://commons.wikimedia.org/wiki/File:Dreissena_polymorpha3.jpg); (b) active consumer (western mosquitofish, https://commons.wikimedia.org/wiki/File:Mosquitofish.jpg); (c) ambush predator (*Enallagma* sp. damselfly larva, https://www.macroinvertebrates.org). Select examples of different cercariae known to be consumed. (d) Small-bodied cercaria (*Plagiorchis* sp.); (e) large-tailed cercaria (magnacauda morphotype); (f) large-bodied cercaria (*Ribeiroia ondatrae*).

The size, morphology, behaviour, density, seasonality and daily emergence patterns of cercariae vary among different trematode species (Combes *et al*., 1994; Esch *et al*., 2002; Koehler *et al*., 2012; Théron, 2015). For instance, while most cercariae have distinct body and tail regions, those of some species have no tail while others have tails that are many times larger than their body (Koehler *et al*., 2012). Such differences are reflected in the wide range in overall cercariae size – some species (e.g. schistosomes) can be as small as 260 μm long (Braun *et al*., 2020), whereas just the tail of ‘magnacauda’ morphotypes can reach 3–4 mm in length (Galaktionov & Dobrovolskij, 2003) – see fig. 2. Cercariae of different species also display distinct diurnal or nocturnal emergence patterns (Hannon *et al*., 2018). As the majority of trematode species are aquatic, cercariae are typically motile and require a second intermediate host (or substrate) in/on which to encyst (Esch *et al*., 2002); ingestion of the latter by a suitable definitive/final host results in successful life cycle completion.

Depending on their environment and their next target host, cercariae may actively or passively move about (i.e. crawling or swimming vs. planktonic drifting) and may be aggregated in space and time within particular microhabitats to maximize the chances of host encounter (Combes *et al*., 1994; Galaktionov & Dobrovolskij, 2003). Some types of cercariae (e.g. Family Azygiidae) even try to attract predators by clustering or mimicking prey movements as they require ingestion as part of their infection process and are often also conspicuous by virtue of their large size (Combes *et al*., 1994). However, cercariae of some aquatic species (e.g. *Fasciola* spp.) encyst upon vegetation, or directly penetrate definitive hosts (e.g. schistosomes), and thus have two – instead of three – host life cycles (fig. 1), while other cercariae (e.g. *Halipegus* spp.) are immobile, remaining at the bottom until an appropriate host is encountered (Esch *et al*., 2002). In addition, cercariae of terrestrial trematodes (e.g. *Dicrocoelium* spp.) can be excreted by their snail hosts within a ‘slime ball’ (Tarry, 1969).

While the consumption of miracidia has been well-documented, along with the corresponding consequences such as reduced transmission (e.g. El Bardicy *et al*., 2009), we focus on cercariae here for two key reasons. As described above, there is considerable interspecific variation among cercariae for traits relevant to the probability of consumption (behaviour, morphotype, etc.), more so than for miracidia. Knowledge gleaned from studies of zooplankton–predator interactions based on these traits is thus likely more applicable to cercariae consumption (see McDevitt-Galles *et al*., 2021) than that of miracidia. Secondly, studies of cercariae consumption reflect a broader coverage of trematode taxa given that those of miracidia consumption have largely focused on schistosomes. Studies with cercariae also span a range of approaches, from laboratory investigations to fieldwork.

### Design of cercariae consumption studies

It is critical to note that our understanding of cercariae consumption to date is inherently tied to study design and methodology. For instance, detecting how consumers respond to cercariae often requires manipulative experiments that use a range of cercariae densities. There is also an inherent trade-off between the realism of conducted experiments and their precision in estimating particular response functions. Thus, the outcome of studies performed at small scales or with simple designs can limit our ability to extrapolate to more natural environments. The appropriate design will often depend on the question(s) of interest. As an example, a study asking whether a given predator eats cercariae can be set up differently from one asking how often, or how many cercariae, does a given predator consume. Investigations of cercariae consumption thus represent a wide range of approaches and methodologies, affecting the conclusions that can be drawn for different systems, as well as influences and implications. Such considerations are the focus of Box 1.

### Studies of cercariae consumption

To summarize the reports of cercariae consumption to date, we conducted a literature search using the database Web of Science in May 2022 for all years with the following search term for titles, abstracts and key words: cercaria* AND (predat* OR consum* OR ingest*). This reflects that consumption and ingestion are not equivalent as the latter does not involve digestion to extract energy and nutrients; however, both have been reported for cercariae and both have potential epidemiological and/or ecological consequences. We excluded cercariae that can survive predation, or require it for transmission (e.g. *Zygocotyle* spp.) as our focus here is on the effects of consumption that inherently involve death of cercariae. We also excluded removal of cercariae from the environment without ingestion/consumption, for example, by getting entangled in bodily structural elements or mucus of non-hosts, failed infection attempts of wrong hosts, etc. (Thieltges *et al*., 2008b), even though this can result in similar community-level consequences for transmission. Based on information in the papers from this literature search, as well as other studies cited by these papers, we summarize the range of cercariae consumers, as well as the consumed cercariae species/types, in table 1.

## Consumers of trematode cercariae

Cercariae of many trematode species can be considered as part of the wider zooplankton community and thus are likely to be consumed by many of the same predators (Morley, 2012). Given the interest in using predators for the biological control of infectious diseases (e.g. schistosomiasis), published investigations of cercariae consumption go back over 75 years (e.g. Oliver-Gonzalez, 1946). Many features of cercariae make them potentially vulnerable to predation, in some cases even more so than zooplankton. Their size is similar to that of zooplankton (roughly 0.2–2000 μm – Moloney & Field, 1991; Hansen *et al*., 1997), and they can also be widely distributed throughout the water column (Haas *et al*., 2008). Unlike many zooplankton, cercariae lack defences (e.g. spines), and may be easier to digest given the absence of a carapace (Johnson *et al*., 2010; McKee *et al*., 2020). These features and others suggest that cercariae fall into a common prey pool and could even represent especially attractive prey items given their high lipid and glycogen content (Lawson & Wilson, 1980; McKee *et al*., 2020).

**Fig. 3.**
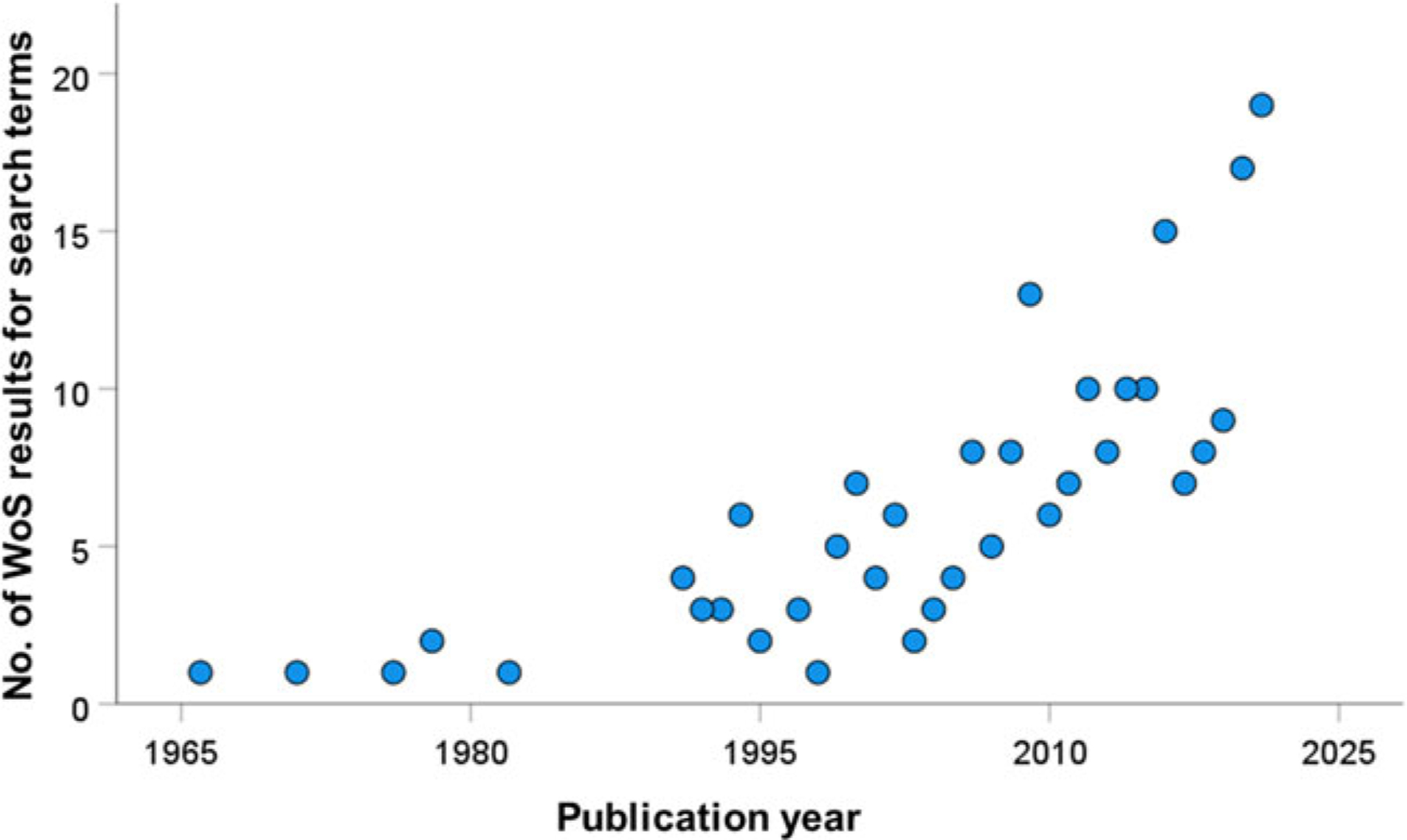
Web of Science results for all years (excluding 2022 as it was incomplete at the time) for the search term ‘cercaria* AND (predat* OR consum* OR ingest*)’.

In our search for studies of cercariae consumption, we found a general increase in such investigations over time, particularly the past 15 years (fig. 3). In total, we documented 60 unique species of consumer (table 1), including 16 vertebrate species (15 of which were fish), and 44 invertebrate species (six insect, 21 crustaceans, nine molluscs, three cnidaria and five others). Of these consumers, 40 represented freshwater species, with the remainder from marine/brackish habitats. There were 121 unique consumer-by-cercaria combinations (78 freshwater, 43 marine/brackish), but these were dominated by four trematode species. Notably, three trematode genera accounted for 28/78 (35%) of the freshwater combinations (*Schistosoma* = 10, *Ribeiroia* = 10 and *Echinostoma* = 7), with *Himasthla* spp. representing 19/43 (44%) of the marine/brackish combinations. The relatively large size of these most-commonly used cercariae in experimental settings may reflect the ease of their observation and enumeration. Overall, the cercariae of 35 different trematode species (25 freshwater and ten marine/brackish) have had at least one consumer reported.

While we do not include such information in table 1, it is important to note that some species did not consume the cercariae with which they were tested (e.g. adult diving beetles in Schotthoefer *et al*., 2007; barnacles in Hopper *et al*., 2008; various consumers in Orlofske *et al*., 2012). As such, cercariae consumption may be rare or non-existent for certain aquatic animals, but we should also not assume that all species of a given taxon will consume cercariae if this has only been demonstrated for a few related consumers within it. For instance, even within the same family, larval dragonflies of some species will readily consume cercariae, but those of other species will not, or will only do so when they are at small body sizes. It is thus important to consider each species (and even ontogenetic stages) separately as they often vary in key traits such as foraging strategy (McDevitt-Galles *et al*., 2021).

## Influences on cercariae consumption

**Fig. 4.**
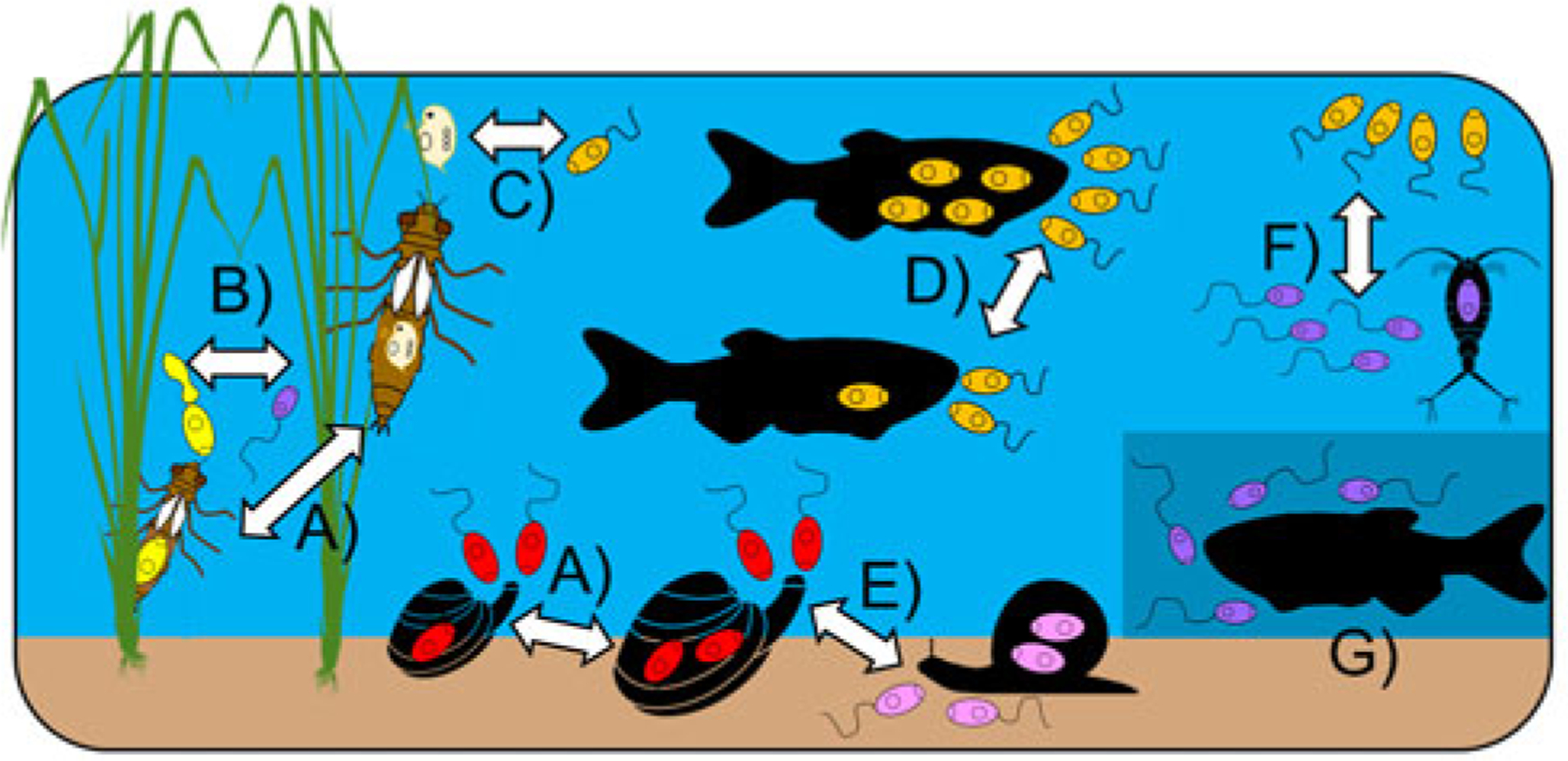
Some known abiotic and biotic influences on the consumption of trematode cercariae. (a) Consumer size (cercariae choice and amount consumed); (b) overall, relative body and tail size of cercariae; (c) alternate prey availability; (d) cercariae density; (e) behaviour of cercariae (swimming vs. crawling) and of predators (passive vs. active consumption); (f) vertical position of cercariae in the water column; (g) intensity of available light. Consumption of various cercariae (by colour) is shown depending on the influence of focus.

The studies conducted to date demonstrate that key aspects of cercariae and consumer behaviour/morphology influence their likelihood of both encounter and subsequent consumption/ingestion (figs 2 and 4). Influential traits of consumers include body size and foraging/hunting strategy. For instance, Welsh *et al*. (2019) reported a positive relationship between cercariae removal rate and oyster or cockle body size, which is likely driven by the fact that larger bivalves filter more water. In other cases, body size of the consumer can negatively influence consumption rate. Catania *et al*. (2016) and McDevitt-Galles *et al*. (2021) found that small-bodied larval odonates generally consumed more cercariae, likely due to either interspecific or ontological differences in body size. Such size-dependent predation may be driven by the ability of predators to detect and handle cercariae, but also optimal foraging decisions when alternative prey are present. However, body size effects are also intertwined with the foraging/hunting strategies employed by consumers. For instance, Welsh *et al*. (2019) found that cercariae removal rates not only depended on consumer size, but also whether they were passive or predatory feeders. Even for very similar predators, feeding strategy is likely influential. For example, the effect of larval odonate body size on cercariae removal varied among predator taxa, with those employing ambush-style tactics consuming more cercariae than active foragers given that the former is optimal for smaller predator–prey size ratios (McDevitt-Galles *et al*., 2021).

When it comes to cercariae features, their overall size, relative tail size and behaviours all influence their likelihood of consumption, as well as the identities of consumer(s) likely to feed on them (figs 2 and 4). Trematode species with large-bodied cercariae or relatively large tails are the most vulnerable to predation by fish and aquatic insects (Orlofske *et al*., 2015; McDevitt-Galles *et al*., 2021). Cercariae behaviour also plays a key role, with both motion type and microhabitat preference affecting consumption. Selbach *et al*. (2019) found that a filter-feeding clam removed free-swimming cercariae, but not cercariae found on sediment, with the reverse seen for vulnerability to a grazing snail predator. Larger cercariae, and more active ones, are probably easier to detect by visual predators and those with large tails may represent a more valuable prey item given that this is the site of glycogen storage (McDevitt-Galles *et al*., 2021). Importantly, however, predators may have circadian patterns of activity that differ from those of vulnerable cercariae, suggesting that temporal overlap in activity should also be taken into account. Because cercariae consumption is so heavily influenced by both parasite and consumer traits, the rate of cercariae removal cannot be generalized, nor easily extrapolated from one trematode–consumer combination to another unless they show strong similarities in key aspects. Predictive frameworks for cercariae consumption will thus require detailed information for multiple parameters, such as those highlighted above.

The consumption of cercariae may also depend on their abundance, which can be described by consumer functional responses as seen with other prey, that is, the feeding rate (response) of a given consumer can vary with prey density (e.g. Oaten & Murdoch, 1975; Dawes & Souza, 2013). This has particularly important implications for consumer interference with transmission at low cercariae densities. Notably, consumers with a Type II response have a high rate of intake at low prey densities before quickly decelerating and reaching a plateau (saturation) at medium prey density (e.g. Holling, 1959; Jeschke *et al*., 2002). In contrast, consumers with Type I functional responses ingest prey at a linear rate irrespective of prey density. This is often seen with filter-feeders whose consumption rate is not constrained by the time required to search for, or handle, prey. Consumers with a Type II response could thus potentially remove more cercariae at low densities than those with Type I response (Born-Torrijos *et al*., 2020), with the latter more effective at removing cercariae in high-density situations given the lack of a plateau. In a Type III response, the rate of prey consumption actually accelerates with increasing density before reaching saturation. This can be explained by a predator learning through greater experience with any prey type, as well as switching to a more common prey item if the density of another decreases (Oaten & Murdoch, 1975).

To date, only a few studies have considered the relationship between cercariae density and probability of consumption. A Type I response was recently reported for a freshwater amphipod consuming cercariae of *Apatemon* sp., but a Type II response (saturation) was seen for this same amphipod when consuming *Diplostomum* sp. cercariae (Born-Torrijos *et al*., 2020). This may be driven by both consumer and parasite behaviour; the intermediate water-column position in which continuously moving *Apatemon* sp. cercariae are found may facilitate constant consumption by benthic-dwelling visual predators, compared to *Diplostomum* cercariae that are often found closer to the water’s surface (Born-Torrijos *et al*., 2020). Another recent study (Born-Torrijos *et al*., 2021) with stickleback predators found a Type II response for consumption of *Plagiorchis* spp. cercariae and a Type III response for those of *Trichobilharzia franki*. As the latter indicates the potential for prey-switching, predators could possibly exploit cercariae with different temporal availabilities (Born-Torrijos *et al*., 2021).

But again, extrapolations among different systems may be difficult, as illustrated by the findings of Welsh *et al*. (2017), who found no cercariae intake saturation points (i.e. a Type I response) for four different marine predators (including two filter-feeders) across the cercariae densities used. This raises an interesting point about the general importance of cercariae (and other free-living infectious stages) as a prey resource for different consumers within a food web. For instance, while low in the food chain, large-bodied filter-feeders may consume large quantities of cercariae because searching and handling time are not constraints, whereas there is usually a strong link between prey size and prey-catching apparatus in predators. At some point, prey may be too small or large to be consumed, suggesting that matches/mismatches between prey and prey-catching mechanics may be more important than consumer trophic position when it comes to cercariae as a food source.

The extent to which cercariae are a food source for predators additionally depends on the presence of alternative prey (fig. 4), although relatively few studies have considered this. Some predators continue to consume the same amount of cercariae if given the choice of alternate prey (Schotthoefer *et al*., 2007; Orlofske *et al*., 2012; Hopkins *et al*., 2013; Welsh *et al*., 2017), or even prefer to feed on cercariae (Catania *et al*., 2016; Mironova *et al*., 2020). This likely reflects optimal foraging, but also depends on the prey options presented, especially their relative sizes and the size of the predator. For instance, small-bodied dragonfly larvae consumed many cercariae, but large-bodied individuals preferred the bigger *Daphnia* offered as alternative prey (Catania *et al*., 2016).

Physical (i.e. abiotic) factors have also been found to influence cercariae consumption. Goedknegt *et al*. (2015) observed that barnacles caused a greater reduction in cercariae transmission to mussels at higher temperatures; this was not seen for oysters or crabs, but mussel infectivity patterns reflected the known thermal responses of the three cercariae predators’ feeding rates. However, the effect of a freshwater mussel on infection transmission to fish was constant in the temperature range (15–23°C) tested by Gopko *et al*. (2020) and temperature did not affect amphipod consumption of various cercariae (Born-Torrijos *et al*., 2020). Orlofske *et al*. (2015) found that far fewer cercariae were consumed during trials conducted in the dark, particularly for trematodes with cercariae <1000 μm in length. Most studies have not considered abiotic influences, or have only examined one at a time, leaving much potential for further investigations of this nature. This is especially critical when considering the implications of cercariae consumption (discussed below) and how this may play out in real-world situations.

## Individual-level implications of cercariae consumption

When we consider the demonstrated, and potential, consequences of consuming cercariae, these can be categorized into those pertaining to individual consumers (e.g. as a viable source of energy or nutrients) and those for the wider community (e.g. reduced transmission).

The sheer biomass of cercariae, sometimes collectively constituting more than that of free-living taxa such as birds or aquatic insects (Kuris *et al*., 2008; Preston *et al*., 2013), could make these a substantial food item for certain consumers, especially small-bodied predators and filter-feeders. Cercariae may be particularly important as food for predators early in ontogeny when their relative sizes present a more optimal match (Catania *et al*., 2016; McDevitt-Galles *et al*., 2021). Apart from biomass, cercariae also exhibit a high productivity, that is, turnover rates of biomass production, as they are usually continuously produced in infected hosts over the transmission season. The latter can be year-round in tropical regions (Cannon, 1979; Aeby, 2007), or confined to the warmer months in regions with more pronounced seasonality (Thieltges & Rick, 2006; Nikolaev *et al*., 2021). The resulting annual production values can be substantial (Kuris *et al*., 2008; Thieltges *et al*., 2008c; Preston *et al*., 2013; Soldánová *et al*., 2016; Rosenkranz *et al*., 2018), such that cercariae may constitute a relatively stable and constantly replenishing resource for consumers.

Cercariae also contain glycogen and lipids (Lawson & Wilson, 1980; Schariter *et al*., 2002), including essential fatty acids (Smith *et al*., 1966; McKee *et al*., 2020). The latter can even occur in similar proportions to those found *Daphnia* spp. – keystone freshwater zooplankton (McKee *et al*., 2020). Lacking the carapaces and defencive spines characteristic of many zooplankton, cercariae might be easier to digest, adding to their energetic value (Johnson *et al*., 2010). As well as their seemingly low investment in anti-predator defences, cercariae could be relatively easy to catch because those of many species display conspicuous swimming behaviours and are usually slower than zooplankton, or even immobile at times (Mironova *et al*., 2020). However, their relative transparency, as well as nocturnal emergence for some species, may make cercariae difficult to detect for visual predators in certain conditions. Given the negligible prey search and handling time for filter-feeders, cercariae could be a particularly profitable prey item for such consumers.

The extent to which parasite consumption can broadly support dietary needs is not well-understood (Johnson *et al*., 2010) and there have been few studies to date that have evaluated consumer performance on diets of cercariae. Those that have been conducted indicate that cercariae can serve as a viable food source. Hopkins *et al*. (2013) found a rapid numerical response by episymbiotic oligochaetes that dwell in the mantle cavities of snails and predate upon cercariae. This response was dependent on cercariae density, with up to 60% oligochaete population growth in just five days (Hopkins *et al*., 2013). Mironova *et al*. (2019) found that reproduction in cyclopoids fed cercariae was not affected over time, suggesting that their dietary needs were met. Not only did McKee *et al*. (2020) report that cercariae and *Daphnia* spp. both contained similar proportions of essential fatty acids, but they found no difference in growth or lipid profiles for larval dragonflies fed experimental diets of either prey item. It is also possible that cercariae might represent a superior food source in certain cases. For instance, Schultz & Koprivnikar (2021) found that benthic oligochaetes given experimental diets that represented mass equivalents of either dead *Daphnia pulex* or cercariae actually exhibited more growth with the latter diet. However, it cannot be assumed that cercariae consumption is always beneficial, or at least neutral. Mironova *et al*. (2019) found that the mortality of certain zooplankton was high (25–28% of individuals) when exposed to cercariae, as these clogged the filtration apparatus of cladocerans and caused internal injuries in predatory rotifers.

## Community-level implications of cercariae consumption

While cercariae ingestion can affect individual consumers as described above, their removal from the environment also has implications for other members of the community, including those related to transmission success, interspecific interactions and perhaps even nutrient dynamics. That non-host consumers could reduce parasite transmission was identified early on as a key consequence stemming from this type of biotic interference (e.g. Rowan, 1958; reviews by Thieltges *et al*., 2008b, 2013; Johnson *et al*., 2010) and the general ability of predators to affect infectious disease dynamics has now been broadly recognized and documented (see reviews by Lopez & Duffy, 2021; Shaw & Civitello, 2021). The use of non-host predators for biocontrol thus holds much potential as a strategy to manage certain infectious diseases (Burge *et al*., 2016; Lopez & Duffy, 2021; Shaw & Civitello, 2021).

Studies of cercariae consumption by non-hosts have provided strong support for interrupted transmission to downstream hosts. This is because host cyst (metacercariae) burdens are usually dose-dependent, that is, the more cercariae a host is exposed to, the more metacercariae will establish in/on it (Karvonen *et al*., 2003; Liddell *et al*., 2017). This in turn means that any reduction in cercariae numbers is likely to reduce infections in downstream hosts. This effect of non-host removal of cercariae has been shown with laboratory experiments, mesocosms and analyses of natural infection patterns (table 1). Effective reduction of host infection can occur through active predation upon cercariae, or their passive consumption or ingestion (i.e. by filter-feeders).

Predation upon cercariae by both vertebrates and invertebrates can reduce transmission. For instance, Orlofske *et al*. (2012) reported that consumption of *Ribeiroia ondatrae* cercariae by larval damselflies caused an approximately 50% reduction in the number of cysts seen in laboratory-exposed tadpoles. Among the examples with fish, guppies (*Lebistes reticulatus*) have been experimentally shown to reduce the transmission of *Schistosoma mansoni* to mice by predating upon cercariae (Christensen, 1979). Cercariae removal by filter/suspension feeders can also effectively reduce transmission, with cockles harbouring fewer cysts of the marine trematode *Himasthla elongata* when in the presence of soft-shelled clams (Thieltges *et al*., 2008a).

However, consumption of cercariae does not always reduce downstream transmission (e.g. Studer *et al*., 2013) and the extent of impeded transmission by non-host consumption may vary among trematode species. For instance, because some trematode species, such as those in the family Hemiuridae, depend on consumption of their cercariae by suitable second intermediate hosts (e.g. aquatic insects or crustaceans) for successful transmission (Schell, 1970), they may be particularly attractive to such consumers. If these cercariae are also more vulnerable to consumption by non-hosts, then their transmission would be disproportionately affected compared to other trematode species with different life cycles. Notably, Hobart *et al*. (2022) recently demonstrated the importance of transmission mode for infection of first intermediate snail hosts by other trematode infectious stages (eggs and miracidia). Commensal oligochaetes protected snails from infection by trematode species with free-swimming miracidia by predating upon them, but did not protect from infections that are acquired when snails ingest trematode eggs in the sediment, emphasizing the importance of parasite–consumer encounters.

Outside of reduced transmission, there are other community-level aspects of cercariae consumption to consider. Nutrient-wise, nitrogen cycling in freshwater systems can be altered by trematode infection of snails (e.g. Mischler *et al*., 2016), but cercariae could also serve as a ‘vector’ by which key compounds are transported (Babaran *et al*., 2020). For example, McKee *et al*. (2020) suggested that cercariae may represent a previously undocumented means by which essential fatty acids are transferred up the food chain to consumers at higher trophic levels, as occurs with other zooplankton. Cercariae could thus provide pathways for energy and nutrient transfer among aquatic organisms that are unlikely to be trophically linked in other ways, such as the planktonic predators of cercariae and the benthic snails from which they emerge (McKee *et al*., 2020; Mironova *et al*., 2020). This may also represent another aquatic–terrestrial link for nutrients, as would occur with fatty acid transfer via emergent insects that consumed cercariae while aquatic larvae (McKee *et al*., 2020). Although the focus to date has been on pelagic consumers of infectious stages, cercariae could also represent substantive energy and nutrient inputs to the benthic community (especially detritivores and microbes) if most die and sink. Notably, Schultz & Koprivnikar (2021) found that most cercariae-derived carbon ended up in sediment-dwelling oligochaetes within experimental mesocosms. In fact, even live cercariae that successfully form cysts in/on their second intermediate hosts could provide a nutritional and energetic benefit to benthic consumers as they often drop their tails when doing so (Esch *et al*., 2002), thus discarded tails may be a potential food source.

Cercariae can also affect predation upon other members of the zooplankton community, particularly if certain predators preferentially feed on cercariae over other zooplankton (e.g. Catania *et al*., 2016). Schultz & Koprivnikar (2019) found that the presence of cercariae promoted higher numbers of *Daphnia* in experimental mesocosms, likely by acting as alternate prey for larval odonates and relieving predation pressure on the cladocerans. As noted above, cercariae could be a particularly important food source for small-bodied or juvenile/larval animals such as fish and aquatic insects because they are relatively small and easy to handle, and this could have population-level or community-level implications by reducing intraspecific and interspecific competition for other prey.

## Gaps in knowledge and future directions

Not only has the importance of consumption been demonstrated for trematode cercariae in the context of energetics and transmission, but also for other parasites such as chytrid fungi. While zooplankton predation upon chytrid zoospores reduces transmission to susceptible phytoplankton, it can also enhance zooplankton population growth by providing an additional resource termed the ‘mycoloop’, which may indirectly reduce populations of non-host phytoplankton by intensified resource competition (Kagami *et al*., 2014). Investigations of cercariae consumption thus have importance not only for better understanding trematode roles in food webs, as well as the relevance of their interactions with non-host organisms, but are also broadly applicable to other parasites and pathogens with free-living infectious stages. As such, the gaps in knowledge and suggestions for future directions, that we identify below may inform work with different systems.

### Consumers of cercariae

While a number of cercariae consumers have been identified so far, there is still a strong need for more studies of this type to better understand the range of taxa capable of this. Considering the six feeding guild categories included in table 1, most investigations of cercariae consumption have involved predators (22 species) and filter/suspension feeders (16 species). Few grazers/scrapers or deposit feeders have been examined in this context (five species total), as is also the case for scavengers/opportunists (six species). As the vast majority of cercariae biomass likely ends up in the benthos, it will be critical to establish its fate, including ingestion by non-predatory consumers.

Cercariae consumption studies have largely focused on non-host species, but it will also be important to test whether potential second intermediate hosts can ingest cercariae capable of infecting them, thereby reducing transmission to themselves (excluding cercariae of species that require predation for infection). This was seen by Orlofske *et al*. (2015), who observed that mosquitofish could reduce their infection load by consuming cercariae to which they were susceptible, but it is a possibility that requires more study. Understanding the extent to which infectious stages are eaten by potential hosts is especially significant if the topological approach continues to be used in constructing food webs – this would mean that a given parasite could be linked to a second intermediate host as both a predator and prey item. To identify possible cercariae consumers, it may be useful to employ some of the theory and empirical work regarding predator and prey traits developed by zooplankton researchers to predict consumption. A few studies have treated cercariae as another zooplankter when trying to understand overall consumption patterns by invertebrates and some small vertebrates (e.g. McDevitt-Galles *et al*., 2021) and this should be further explored.

A striking take-away from table 1 is that all examples of cercariae consumption so far come from aquatic systems. There are admittedly fewer terrestrial species of trematode, but their cercariae (and associated products) could still be consumed by non-hosts. For example, *Dicrocoelium* cercariae are found in a slime ball and are transmitted when eaten by ants (Manga-González et al., 2001), making it possible that non-hosts also encounter and consume these. Other genera in the family Dicrocoeliidae also rely on ingestion of cercariae by arthropod second intermediate hosts and even deposit cercariae on vegetation, sometimes in masses (Schell, 1970). Similarly, cercariae of some trematode species within the family Brachylaemidae creep out onto the surface of their terrestrial first intermediate snail host and are transmitted to another snail through contact (Schell, 1970), also making them vulnerable to consumption if dislodged. In addition, cercariae consumption studies to date have almost exclusively focused on the consumption of live cercariae. This almost certainly underestimates the energetic and nutritional contributions of cercariae (and other infectious stages) to food webs given that many will die and end up in the benthos, an issue discussed further below.

Feeding experiments have been a valuable approach to identifying cercariae consumers, but favour work with taxa that can be relatively easily acquired and studied in laboratory or mesocosm settings. This makes it difficult to recognize cryptic consumers, as well as those of dead rather than living cercariae. Given the high production of cercarial stages, it is likely that many of them will not infect hosts, nor be ingested by consumers in the water column, but die and sink out of the latter to become available for microbial and detritivore consumption. In addition, the high production of bottom-dwelling cercariae by many trematode species (e.g. Rosenkranz *et al*., 2018) might mean that their energy is primarily available to benthic consumers. This form of cercarial consumption is largely unstudied to date, but it is likely that the contribution of dead cercariae to energy flow through the detritivore and microbial compartments of food webs is substantial. Identifying and quantifying such consumption of cercariae will require appropriate methodology.

Manually counting the remaining cercariae after consumer exposure lends itself well to small-scale experiments with low volumes of water and/or cercariae numbers, but not to larger and more complex studies involving multiple taxa at once, especially relatively cryptic consumers (e.g. detritivores). It is therefore critical to develop and refine tools to trace cercariae consumption and the fate of cercariae biomass, such as use of stable isotope markers, fluorescing dyes, or other types of tags. As an example, Schultz & Koprivnikar (2021) recently demonstrated the use of cercariae labelled with Carbon-13 to track their fate in experimental mesocosms with potential consumers representing different trophic levels. Genetic approaches also hold promise. For instance, while the detection of parasite DNA in faecal material has typically been used to identify infection within a given host population (Bass *et al*., 2023), it could also be used to detect consumption of cercariae by non-hosts (i.e. a positive result would not arise from the presence of other trematode stages such as eggs).

Such approaches and others would allow us to understand the relative and absolute contributions of cercariae as a resource to food web energetics via different pathways such as pelagic and benthic consumption of live cercariae by macrofauna and consumption of dead cercariae by detritivores and microbes. We also need to understand how much trematode cercariae might truly contribute to the diet of consumers (Thieltges *et al*., 2013) – observations from feeding trials may not necessarily reflect what occurs in natural settings. Tools to track cercariae consumption over relatively long periods of time will be useful in this context.

Researchers should strongly consider whether consumption in laboratory settings realistically reflects what occurs in the field under more complex conditions. Small water volumes, as well as lack of environmental complexity (such as submerged vegetation), may exaggerate consumer–cercariae contact rates. Conversely, predators may not behave normally depending on the set-up used, as seen by Schotthoefer *et al*. (2007), who reported erratic swimming by diving beetles and tadpole shrimp when placed in small cell wells that may have distracted them from cercariae prey. Cercariae consumption may also depend on their visibility, and be affected by turbidity or other factors, for example, suspended algae in Schotthoefer *et al*. (2007). Suboptimal testing arenas (see Box 1) could result in certain organisms being mischaracterized as key cercariae consumers through unnaturally high contact rates, but also in ‘false negatives’ if others do not display regular feeding behaviours. In general, we strongly urge future experiments to employ more complex environmental conditions, focusing on factors that are most likely to affect cercariae–consumer encounter, as well as reflecting consumer natural habitats. However, we recognize that there is often a trade-off between experimental complexity/scale and the ability to precisely quantify the fate of administered cercariae, requiring a balanced approach. Such work will be vital for understanding the extent to which certain consumers could be useful in biocontrol of trematode infections, that is, the rate of cercariae removal seen in laboratory settings may not be reflective of that in the field, making extrapolations from the former to the latter inaccurate.

### Factors influencing cercariae consumption

Realistic testing set-ups are necessary to identify cercariae consumers, as well as their removal rates. Beyond the factors noted in Box 1, very few experiments have offered predators an alternative, making it unclear whether they would consume cercariae over other prey. Given that most studies starve predators for at least some time beforehand, hungry predators may eat what is offered to them if it is the only choice. Of the investigations that have included alternative prey such as *Daphnia*, some found that predators generally continued to consume cercariae (Schotthoefer *et al*., 2007; Orlofske *et al*., 2012), but prey preference can also be dependent on body size, with relatively large predators often bypassing small cercariae in favour of larger zooplankton (Catania *et al*., 2016). More feeding studies should consider the diversity of potential prey available in natural settings and how this affects cercariae consumption.

While there is a general need for more studies that examine consumption of cercariae under varied abiotic and biotic conditions, temperature is a particularly important consideration given that every consumer listed in table 1 is an ectotherm or poikilotherm, thus their feeding rates may be affected by changing global temperatures (Kirk *et al*., 2022). Without understanding how temperature affects parasite interactions with both hosts and non-hosts, it will be difficult to predict how climate change may influence infectious disease dynamics (Byers, 2021). For example, climate change may affect the potential for filter-feeders to mitigate disease risk in all scenarios (Burge *et al*., 2016).

Given that four species of trematode (~0.02% of the >20,000 species identified to date; Esch *et al*., 2002) have disproportionately accounted for most studies of cercariae consumption until now, the inclusion of more species will help us to better understand how commonly this occurs, and under which circumstances. For instance, the cercariae of some trematodes might be consumed far more than others if they possess certain traits. Across trematode species, there is a wide range in cercariae size and tail to body ratio (Koehler *et al*., 2012), as well as cercariae microhabitat use and activity patterns (Combes *et al*., 1994). This might make particular cercariae more prone to consumption and can also help to identify potential consumers. Not only is it important to provide zooplankton as alternative prey in feeding experiments, but also other cercariae. In a single freshwater pond, cercariae from as many as 18 different species of trematode can simultaneously occur (Loy & Haas, 2001), meaning that predators likely make choices among these as well; however, this has not yet been tested to the best of our knowledge. Different relative densities of prey should also be included to see if switching occurs considering the varied consumer functional responses to cercariae that have been reported (e.g. Born-Torrijos *et al*., 2020, 2021).

### Individual-level consequences of cercariae consumption

Although we note earlier that consumption and ingestion are not equivalent, this has generally been assumed for studies involving cercariae, that is, the take-up of cercariae by a given organism (ingestion) is followed by internal digestion (consumption). This may be true in the majority of cases, as reflected by our use of terminology referring to consumption throughout, but it is important to verify. For instance, ingestion can still reduce transmission by removing cercariae, but only consumption confers energetic or nutritional benefits by using what cercariae contain. As an example, Gopko *et al*. (2020) found that mussels effectively reduce transmission of cercariae to fish by filtering these out of the water, but appear to expel them as pseudofaeces. Such cercariae are then presumably no longer infective, but this should be confirmed given that other helminth infectious stages (including trematode eggs) can remain intact and viable after ingestion by non-hosts (Morley, 2022). This also has implications for understanding the ultimate fate of cercariae biomass, which would be broken down by the benthic community in this case, not the original organisms ingesting cercariae.

Related to the question of ingestion vs. consumption, there is a strong need for more work that quantifies the energetic and nutritional attributes of cercariae. Cercariae are known to contain glycogen, particularly in their tails (Lawson & Wilson, 1980), as well as lipids (Smith *et al*., 1966), but few studies have attempted quantification (e.g. Schariter *et al*., 2002; Fokina *et al*., 2018), much less combining this information with known estimates of their biomass to assess the contributions to food webs. McKee *et al*. (2020) found that individual cercariae of *R. ondatrae* contain appreciable quantities of essential fatty acids and calculated how much the annual production of these cercariae may contribute to lipid availability in a series of freshwater ponds.

While information of this type is sparse, considering parasites from an energetic perspective could help to integrate them into food webs (Sukhdeo, 2012; Thieltges *et al*., 2013) and should be considered as a priority. Incredibly enough, we do not yet know the caloric content of any cercariae. This is no doubt largely attributable to logistical challenges, including the large numbers of cercariae required for the necessary analytic methods (e.g. ~30,000 cercariae for one aggregate sample of lipids in McKee *et al*., 2020). Quantifying key energetic and nutritional attributes of cercariae will also help in assessing their quality as a food source relative to other zooplankton. Assumptions have been made about their equivalency (e.g. Kaplan *et al*., 2009), but this must be empirically tested. Only a handful of studies have done so thus far, with the overall trend of consumers exhibiting individual-level or population-level growth on diets of cercariae (Hopkins *et al*., 2013) that matches or even exceeds that of consumers fed zooplankton (McKee *et al*., 2020; Schultz & Koprivnikar, 2021).

Although these studies suggest that parasite consumption can broadly support dietary needs of consumers, it remains an open question whether cercarial resources add to the pool of resources for specific consumers or only substitute what otherwise would be provided by the reproductive propagules of their hosts. Trematode infections usually lead to the castration of their snail hosts (Lafferty & Kuris, 2009) which then no longer produce reproductive propagules that are also likely a food resource for many consumers of cercariae. It thus remains to be studied whether the annual production of cercariae is higher than the reproduction of reproductive stages by snail hosts and whether these stages are energetically equivalent and used to the same extent by consumers. Even if these two food resources are energetically similar, they are almost certainly different in other respects – juvenile/larval snails are unlikely to equal cercariae/zooplankton in an overall sense. This will require studies that compare the energetic and nutritional attributes of cercariae with those of the reproductive stages of infected hosts. Beyond caloric or nutrient content, the value of cercariae as a food source will be influenced by their handling time and digestibility, especially in comparison to zooplankton. Future studies should therefore also account for these aspects.

We should additionally consider how bottom-up processes could affect the quality of cercariae as a food source. For instance, changes in primary production could affect the tissue composition of gastropods typically used as first intermediate hosts, thereby influencing the content of the cercariae developing within sporocysts and rediae (parthenitae) within the host’s digestive gonadal gland complex. Given that certain aspects of cercariae composition strongly reflect that of the snails in which they developed (as seen for lipids in Furlong & Caulfield, 1988), the nutritional quality of parasites could vary in space and time depending on host diet. However, Babaran *et al*. (2021) found that the content of nutritionally important polyunsaturated fatty acids (mainly omega-3) in trematode sporocysts was largely consistent even if their snail hosts were fed different diets (cyanobacteria, green algae and diatoms). Intriguingly, trematode-containing snail tissue also generally contained more of these fatty acids relative to snail-only tissue, suggesting that trematodes could be trophic upgraders of such vital lipids (Babaran *et al*., 2021). This reflects another outstanding general question – to what extent does the environment affect the quality of cercariae as a food resource?

### Community-level consequences of cercariae consumption

Reduced transmission is a major potential consequence of cercariae removal from the environment and there has been substantial interest in using cercariae consumers as a form of biocontrol (Lopez & Duffy, 2021; Shaw & Civitello, 2021). This said, most reports of cercariae consumption do not investigate whether this affected transmission to downstream hosts. Of the consumer–cercariae combinations listed in table 1, transmission effects were not studied for 96 of these (note that some combinations are represented by multiple investigations). Meaningful reductions in transmission were seen for 31 of 36 combinations that considered this (i.e. no effects were seen five times); however, separate studies with the same cercaria and consumer sometimes showed different results. For example, consumption of *Himasthla elongata* cercariae by the Pacific oyster (*Crassostrea gigas*) can reduce transmission or have no effect (e.g. Thieltges *et al*., 2008a; Welsh *et al*., 2017). Transmission-related effects of cercariae consumption have been reported for multiple feeding guilds, including deposit feeders, predators, filter/suspension feeders and scavengers/opportunists.

While laboratory and mesocosm studies have demonstrated transmission reductions via cercariae removal, we need more empirical investigations that utilize approaches which will let us determine whether transmission in natural settings would actually differ in the absence of cercariae consumption. While some field studies (e.g. Rohr *et al*., 2015) have suggested a link between trematode infections in second intermediate hosts and the presence of predators (both of cercariae and snails), additional information beyond natural infection loads and consumer/predator richness will help in supporting a causal relationship. For instance, it would be helpful to know something about the density of infected snails (and thus cercariae released) vs. the number of successful infections in second intermediate hosts to calculate their exposure risk to begin with.

It is also unclear whether there is a positive relationship between consumer species richness and overall abundance – is the richness of consumers important, or simply how many ‘mouths’ of any species are removing cercariae? Similarly, does high consumer richness (i.e. number of species) reduce transmission, or does high diversity play a role, such as the presence of consumers across diverse feeding guilds? For larval amphibians and odonates, Koprivnikar *et al*. (2017) found that the density of cercariae predators was a stronger predictor of natural trematode infection loads in these hosts than predator richness. We should therefore especially aim to quantify consumption in a wider range of organisms that span different functional roles and trophic levels. For instance, when it comes to aquatic insects, most investigations have focused on larval odonates (see table 1), although other insects have been tested and found not to consume cercariae (e.g. backswimmers in Orlofske *et al*., 2012), which may discourage further work with certain taxa. Investigations that take into account potential consumers (or merely ingesters) with different ecologies and feeding modes should thus be a priority.

The directions suggested above will help us to better understand the link between cercariae transmission and community biodiversity, which is critical for estimating possible reductions in transmission via cercariae consumption and therefore the possible use of various consumers for biocontrol, as well as priorities for conservation. Importantly, some community members may be keystone predators in this context given that relative cercariae removal rates can vary among species (e.g. Welsh *et al*., 2017) and parasite removal rates in predators can be species-specific (Welsh *et al*., 2019). It is thus possible that the most effective biocontrol is achieved by promoting the abundance of certain consumers. Although it is not easy to compare cercariae removal capacity among different species with very different morphologies or removal mechanisms (Johnson & Thieltges, 2010), quantitative studies will be essential to determine the extent to which cercariae can be removed from the environment in a manner that significantly affects transmission.

There remain many challenges inherent to empirically demonstrating consumption-mediated reductions in transmission, including the question of whether the mortality of cercariae through consumption by consumers is additive or compensatory. Most of the myriad of cercariae emitted from their hosts may die by other means anyway, for example, not finding a downstream host, or detrimental physical or chemical factors in the environment. Small-scale experiments in the laboratory may thus not give a realistic picture regarding the relative relevance of consumer-induced mortality in comparison to other potential mortality factors. Hence, identifying the relative importance of additive and compensatory mortality will be an important future research area.

Good estimates of cercariae consumption will also require a closer examination of basic predator–prey dynamics, including both numerical (population growth) and functional (feeding) responses. As discussed earlier, just as with zooplankton prey, different predators exhibit different functional response curves with increasing cercariae density, which also differs depending with the type of cercaria (e.g. Orlofske *et al*., 2015; Welsh *et al*., 2019; Born-Torrijos *et al*., 2020, 2021; Mironova *et al*., 2020). All three possible consumer functional responses (Types I, II and III) have been reported thus far. Further work of this type is critical because consumer biocontrol ability could strongly depend on their functional response. Certain organisms, such as filter-feeders, may be capable of removing many cercariae at high densities if their consumption does not reach a saturation point (i.e. a Type I response, e.g. Welsh *et al*., 2017; Born-Torrijos *et al*., 2020). However, consumers with Type II responses might be most effective at suppressing cercariae numbers at low prey densities given their relatively high removal rate before their rapid saturation (Born-Torrijos et al., 2021).

It is also important to bear in mind evolutionary pressures. Specifically, if predation has meaningful negative effects on transmission, we might expect selection for cercariae to avoid this in the majority of trematode species. The simple fact that cercariae tend to emerge from their first intermediate hosts in very large numbers may shield them from meaningful reductions in transmission due to non-host consumption. This said, there are also likely trade-offs when it comes to cercariae features that can promote host finding but increase vulnerability to predation, such as large cercariae size and activity level. The synchronization of some cercariae with host time and host space may also make them a rather ‘predictable’ food source for potential predators. Additionally, possible predators could be attracted to infected snails and sit close by to ‘snatch up’ emerging cercariae right at the source. With such vulnerabilities in mind, it is even possible that certain trematode species requiring cercariae ingestion by intermediate hosts for transmission may have taken advantage of accidental occurrences in ancestral lineages if these were frequent and advantageous. These trematodes, along with related species, may thus be particularly vulnerable to consumption by non-hosts, making it easier to identify situations where cercariae consumption may be substantial.

In terms of cercariae-mediated energy and nutrient transfer, recent work has shown that this is possible (e.g. McKee *et al*., 2020), but we need more direct studies that empirically consider this. Some of the directions suggested above for future investigations of individual-level consequences of cercariae consumption will be relevant in this context, such as estimating how many potential calories per year cercariae might represent as a food source in particular habitats. This can be built upon by considering further trophic transfer. For instance, is cercariae energy and nutrient content mostly transferred to primary and secondary consumers, or does much of it end up in the benthos? Of the incredible number of cercariae that can emerge from their hosts, most are obviously unsuccessful at finding a suitable second intermediate host, but their ultimate fate is essentially unknown.

Finally, while there is a strong argument to be made for considering free-living infectious stages as part of the wider zooplankton community (Morley, 2012), this may not necessarily be an appropriate paradigm when it comes to the trophic transfer of energy and nutrients. For example, while parasites do not universally display greater Nitrogen-15 isotope fractionation than their hosts, which would indicate a higher trophic position more akin to that of predator, this varies among taxa, as well as between freshwater and marine systems (Thieltges *et al*., 2019). It is unclear whether certain parasites, or certain life history stages such as cercariae, may differ enough from zooplankton in their composition that they could be more or less efficient in the trophic transfer of energy or compounds. Such foundational information is central to understanding what an ecosystem without trematodes would look like relative to one where they are abundant in terms of energy flow and nutrient cycling. Cercariae may not simply be like other zooplankton in this respect and perhaps the free-living infectious stages of other parasites and pathogens are not either.

## Conclusions

There is now widespread recognition that parasites need to be included in food webs, and we argue that the most essential pathways involve their consumption, especially that of free-living infectious stages such as trematode cercariae. Interest in cercariae consumption has primarily centred around the implications for transmission, but we need more information to understand the potential for using consumers as biocontrols that remove cercariae in natural systems, as has been suggested for those responsible for ‘swimmer’s itch’ (Soldanová et al., 2013; Born-Torrijos et al., 2021). Given the range of cercariae consumers identified thus far, maintaining biodiverse communities with many consumers of different morphologies, sizes and removal mechanisms may be important for limiting trematode transmission success, that is, cercariae consumers could contribute to the biodiversity-dilution effect (Johnson & Thieltges, 2010).

Critically, we do not yet understand the potential magnitude of these effects – it is completely possible that cercariae consumption does not greatly reduce transmission in natural systems, and we need convincing evidence of this, along with having other key transmission-related information (e.g. exposure risk to begin with). Related to this, the diversity of consumers may not be as important as their overall biomass or trophic level, but again, this requires empirical testing. Future studies should consider the range of possible cercariae consumers, the morphotypes and behaviours of cercariae that are most likely to be ingested, how many can be removed when considering realistic densities and which conditions favour cercariae removal. Beyond transmission, there are other potential indirect effects on the free-living community to be considered, especially if cercariae are functionally similar to zooplankton in many ways. Drawing upon theory or empirical work derived from zooplankton interactions between predators and prey might let us forecast forward to better understand interactions between cercariae and their consumers, as well as among cercariae of different types and other planktonic organisms.

It will also be important to conduct further work on the value of cercariae as a food source in themselves, including whether the consumer actually benefits, and how. While we might assume that the primary beneficiaries of cercariae consumption are predators, the largest inputs of cercariae-derived nutrients and energy might be to the benthos. Notably, large quantities of cercariae would still be present in habitats even if their second intermediate hosts are not. The consumption of cercariae in this scenario would have no implications for transmission, but they could potentially be significant as a resource. In the context of ecosystem processes, a recent meta-analysis found that parasites and pathogens had greater effects on primary production than on secondary production or biogeochemical cycles (Fischhoff *et al*., 2020), but this may change if we more carefully consider the consumption of infectious stages such as cercariae given that few studies have quantified such effects (Preston *et al*., 2016).

While we have focused here on trematode cercariae, researchers should also consider the possible ingestion or consumption of other free-living aquatic helminth infectious stages by non-host organisms. Some studies have investigated non-host consumption of trematode miracidia (e.g., Hobart *et al*., 2022), as well as that of monogenean oncomiracidia (e.g. Militz & Hutson, 2015), but there do not appear to be any involving the coracidia of cestodes, or the acanthors of acanthocephalans. Beyond pursuing similar investigations with other helminths, studies with trematode cercariae can also help to guide work with the free-living stages of non-helminth parasites and pathogens and vice versa. For example, fungal zoospores are an excellent source of essential fatty acids for aquatic consumers (Kagami *et al*., 2007), which was then also shown with trematode cercariae (McKee *et al*., 2020). The infectious stages of other parasites and pathogens might thus also represent sources of energy and nutrients, and their consumption may be considerable as well, as recently demonstrated for marine viruses (Welsh *et al*., 2020). Continued work with trematode cercariae will move us towards a better understanding of the broad implications associated with parasite and pathogen consumption, particularly that of their free-living stages.

## Figures and Tables

**Table 1.** Non-host organisms demonstrated to remove trematode cercariae from the aquatic environment.

Demonstrated non-host live cercaria consumer	Feeding guild	Trematode species	Habitat type	Transmission effect	Citation
Fish					
Guppy (*Lebistes reticulatus*)	Omnivore	*Schistosoma mansoni*	FW	N, N, Y, N, Y	Oliver-Gonzalez (1946), Rowan (1958), Pellegrino *et al*. (1966), Knight *et al*. (1970) and Christensen (1979)
Western mosquitofish (*Gambusia affinis*)	Predator	*Ribeiroia ondatrae*	FW	N	Orlofske *et al*. (2012, 2015)
Western mosquitofish (*G. affinis*)	Predator	*Magnacauda* sp.	FW	NS	Orlofske *et al*. (2015)
Western mosquitofish (*G. affinis*)	Predator	*Echinostoma trivolvis*	FW	NS	Orlofske *et al*. (2015)
Western mosquitofish (*G. affinis*)	Predator	*Cephalogonimus* sp.	FW	NS	Orlofske *et al*. (2015)
African killifish (*Epiplatys fasciolatus*)	Predator	*S. mansoni*	FW	NS	Siau *et al*. (1992)
Zebrafish (*Brachydanio rerio*)	Omnivore	*Transversotrema patialense*	FW	Y	Anderson et al. (1978)
California killifish (*Fundulus parvipinnis*)	Predator	*Cloacitrema michiganensis*	MB	NS	Kaplan *et al*.(2009)
California killifish (*F. parvipinnis*)	Predator	*Himasthla rhigedana*	MB	NS	Kaplan *et al*. (2009)
California killifish (*F. parvipinnis*)	Predator	*Himasthla* sp. B.	MB	NS	Kaplan *et al*. (2009)
California killifish (*F. parvipinnis*)	Predator	*Parorchis acanthus*	MB	NS	Kaplan *et al*. (2009)
California killifish (*F. parvipinnis*)	Predator	*Renicola buchanani*	MB	NS	Kaplan *et al*. (2009)
Arrow goby (*Clevelandia ios*)	Omnivore	*C. michiganensis*	MB	NS	Kaplan *et al*. (2009)
Arrow goby (*C. ios*)	Omnivore	*H. rhigedana*	MB	NS	Kaplan *et al*. (2009)
Arrow goby (*C. ios*)	Omnivore	*Acanthoparyphium spinulosum*	MB	NS	Kaplan *et al*. (2009)
Arrow goby (*C. ios*)	Omnivore	*Himasthla* sp. B.	MB	NS	Kaplan *et al*. (2009)
Arrow goby (*C. ios*)	Omnivore	*P. acanthus*	MB	NS	Kaplan *et al*. (2009)
Staghorn sculpin (*Leptocottus armatus*)	Predator	*C. michiganensis*	MB	NS	Kaplan *et al*. (2009)
Staghorn sculpin (*L. armatus*)	Predator	*H. rhigedana*	MB	NS	Kaplan *et al*. (2009)
Staghorn sculpin (*L. armatus*)	Predator	*P. acanthus*	MB	NS	Kaplan *et al*. (2009)
Longjaw mudsucker (*Gillichthys mirabilis*)	Predator	*H. rhigedana*	MB	NS	Kaplan *et al*. (2009)
Longjaw mudsucker (G. *mirabilis*)	Predator	*Himasthla* sp. B.	MB	NS	Kaplan *et al*. (2009)
Diamond turbot (*Hypsopsetta guttulata*)	Predator	*C. michiganensis*	MB	NS	Kaplan *et al*. (2009)
Diamond turbot (*H. guttulata*)	Predator	*H. rhigedana*	MB	NS	Kaplan *et al*. (2009)
Diamond turbot (*H. guttulata*)	Predator	*Himasthla* sp. B.	MB	NS	Kaplan *et al*. (2009)
Diamond turbot (*H. guttulata*)	Predator	*P. acanthus*	MB	NS	Kaplan *et al*. (2009)
Diamond turbot (*H. guttulata*)	Predator	*R. buchanani*	MB	NS	Kaplan *et al*. (2009)
Topsmelt (*Atherinops affinis*)	Omnivore	*C. michiganensis*	MB	NS	Kaplan *et al*. (2009)
Topsmelt (*A. affinis*)	Omnivore	*H. rhigedana*	MB	NS	Kaplan *et al*. (2009)
Three-spined stickleback (*Gasterosteus aculeatus*)	Predator	*Plagiorchis* spp.	FW	NS	Born-Torrijos *et al*. (2021)
Three-spined stickleback (*G. aculeatus*)	Predator	*Trichobilharzia franki*	FW	NS	Born-Torrijos *et al*. (2021)
Rainbow trout (*Oncorhynchus mykiss*)	Predator	*Plagiorchis elegans*	FW	NS	Heinclová (2018)
Roach (*Rutilus rutilus*)	Omnivore	*P. elegans*	FW	NS	Heinclová (2018)
White bream (*Blicca bjoerkna*)	Predator	*P. elegans*	FW	NS	Heinclová (2018)
White bream (*B. bjoerkna*)	Predator	*Echinoparyphium aconiatum*	FW	NS	Heinclová (2018)
Stone moroko (*Pseudorasbora parva*)	Omnivore	*P. elegans*	FW	NS	Heinclová (2018)
Stone moroko (*P. parva*)	Omnivore	*E. aconiatum*	FW	NS	Heinclová (2018)
White bream (*B. bjoerkna*)	Predator	*Trichobilharzia szidati*	FW	NS	Heinclová (2018)
Stone moroko (*P. parva*)	Omnivore	*T. szidati*	FW	NS	Heinclová (2018)
Other vertebrates					
California newt larvae (*Taricha torosa*)	Predator	*R. ondatrae*	FW	Y	Orlofske *et al*. (2012)
insects					
Damselfly larvae (Coenagrionidae)	Predator	*R. ondatrae*	FW	NS	Schotthoefer *et al*. (2007)
Damselfly larvae (Coenagrionidae)	Predator	*Australapatemon sp*.	FW	NS	McDevitt-Galles *et al*. (2021)
Damselfly larvae (Coenagrionidae)	Predator	Brevifurcate-apharyngeate	FW	NS	McDevitt-Galles *et al*. (2021)
Damselfly larvae (Coenagrionidae; *Enallagma* sp.)	Predator	*R. ondatrae*	FW	Y	Orlofske *et al*. (2012, 2015)
Damselfly larvae (Coenagrionidae; *Enallagma* sp.)	Predator	*Magnacauda sp*.	FW	NS	Orlofske *et al*. (2015)
Damselfly larvae (Coenagrionidae; *Enallagma* sp.)	Predator	*E. trivolvis*	FW	NS	Orlofske *et al*. (2015)
Damselfly larvae (Coenagrionidae; *Enallagma* sp.)	Predator	*Cephalogonimus sp*.	FW	NS	Orlofske *et al*. (2015)
Damselfly larvae (Lestidae)	Predator	*Australapatemon sp*.	FW	NS	McDevitt-Galles *et al*. (2021)
Damselfly larvae (Lestidae)	Predator	Brevifurcate–apharyngeate	FW	NS	McDevitt-Galles *et al*. (2021)
Damselfly larvae (Lestidae)	Predator	*Echinostoma sp*.	FW	NS	McDevitt-Galles *et al*. (2021)
Damselfly larvae (Lestidae; *Lestes* sp.)	Predator	*R. ondatrae*	FW	Y	Orlofske *et al*. (2012)
Dragonfly larvae (Libellulidae)	Predator	*R. ondatrae*	FW	NS	Schotthoefer *et al*. (2007)
Dragonfly larvae (Libellulidae)	Predator	*Cephalogonimus americanus*	FW	NS	McDevitt-Galles *et al*. (2021)
Dragonfly larvae (Libellulidae)	Predator	Brevifurcate–apharyngeate	FW	NS	McDevitt-Galles *et al*. (2021)
Dragonfly larvae (Libellulidae)	Predator	*Australapatemon* sp.	FW	NS	McDevitt-Galles *et al*. (2021)
Dragonfly larvae (Libellulidae; *Leucorrhinia intacta*)	Predator	*Cotylurus sp*.	FW	NS	Catania *et al*. (2016)
Dragonfly larvae (Libellulidae; L. *intacta*)	Predator	*Posthodiplostomum* sp.	FW	NS	Catania *et al*. (2016)
Dragonfly larvae (Libellulidae; L. *intacta*)	Predator	*Plagiorchis* sp.	FW	NS	Schultz & Koprivnikar (2019)
Dragonfly larvae (Libellulidae; L. *intacta*)	Predator	*R. ondatrae*	FW	NS	McKee *et al*. (2020)
Dragonfly larvae (Libellulidae; *Erythemus simplicicolis*)	Predator	*E. trivolvis*	FW	NS	Rohr *et al*. (2015)
Dragonfly larvae (Libellulidae; *Sympetrum semicinctum*)	Predator	*E. trivolvis*	FW	Y	Rohr *et al*. (2015)
Dragonfly larvae (Aeshnidae; *Anax junius*)	Predator	*E. trivolvis*	FW	NS	Rohr *et al*. (2015)
Dragonfly larvae (Aeshnidae)	Predator	*Australapatemon* sp.	FW	NS	McDevitt-Galles *et al*. (2021)
Dragonfly larvae (Aeshnidae)	Predator	Brevifurcate–apharyngeate	FW	NS	McDevitt-Galles *et al*. (2021)
Crustaceans					
Branchiopod (*Daphnia pulex*)	Filter/suspension	*S. mansoni*	FW	Y	Christensen (1979)
Branchiopod (*Daphnia longispina*)	Filter/suspension	*S. mansoni*	FW	Y	Christensen (1979)
Branchiopod (*Cyzicus californicus*)	Filter/suspension	*R. ondatrae*	FW	NS	Orlofske *et al*. (2012)
Branchiopod (*Sida crystallina*)	Filter/suspension	*Diplostomum pseudospathaceum*	FW	NS	Mironova *et al*. (2019)
Ostracod (*Notodromas monacha*)	Scavenger/opportunist	*S. mansoni*	FW	Y	Christensen (1979)
Ostracod (*Cypria ophthalmica*)	Scavenger/opportunist	*S. mansoni*	FW	Y	Christensen (1979)
Ostracod (*Candonocypris novaezelandiae*)	Scavenger/opportunist	*S. mansoni*	FW	NS	Yousif *et al*. (2013)
Ostracod (*Cypridopsis hartwigi*)	Scavenger/opportunist	*S. mansoni*	FW	NS	Courmes *et al*. (1964)
Hexanauplid (Cyclopoida)	Omnivore	*R. ondatrae*	FW	NS	Schotthoefer *et al*. (2007)
Hexanauplid (*Megacyclops viridis*)	Omnivore	*D. pseudospathaceum*	FW	NS	Mironova *et al*. (2019)
Hexanauplid (*Macrocyclops distinctus*)	Predator	*D. pseudospathaceum*	FW	NS	Mironova *et al*. (2019)
Hexanauplid (*Mesocyclops leuckarti aequatorialis*)	Predator	*Schistosoma incognitum*	FW	NS	Banerjee (1996)
Hexanauplid (*M. leuckarti aequatorialis*)	Predator	*Fasciola gigantica*	FW	NS	Banerjee (1996)
Hexanauplid (*Mesocyclops leuckarti*)	Predator	*Opisthorchis viverrini*	FW	NS	Intapan *et al*. (1992)
Hexanauplid (*Arctodiaptomus paulseni*)	Omnivore	*D. pseudospathaceum*	FW	NS	Mironova *et al*. (2019)
Malacostracid (crab - *Carcinus maenas*)	Predator	*Himasthla elongata*	MB	Y	Thieltges *et al*. (2008a)
Malacostracid (crab - *Hemigrapsus takanoi*)	Predator	*H. elongata*	MB	Y, Y	Welsh *et al*. (2014, 2017)
Malacostracid (shrimp - *Crangon crangon*)	Predator	*H. elongata*	MB	NS, NS, Y	Welsh *et al*. (2014, 2017) and Thieltges *et al*. (2008a)
Malacostracid (amphipod - *Gammarus marinus*)	Scavenger/opportunist	*H. elongata*	MB	NS	Welsh *et al*. (2014)
Malacostracid (amphipod - *G. lacustris*)	Scavenger/opportunist	*Diplostomum sp*.	FW	NS	Born-Torrijos *et al*. (2020)
Malacostracid (amphipod - *G. lacustris*)	Scavenger/opportunist	*Apatemon sp*.	FW	NS	Born-Torrijos *et al*. (2020)
Malacostracid (amphipod - *G. lacustris*)	Scavenger/opportunist	*Trichobilharzia* sp.	FW	NS	Born-Torrijos *et al*. (2020)
Malacostracid (amphipod - *Dikerogammarus villosus*)	Predator	*Trichobilharzia* sp.	FW	NS	Stanicka *et al*. (2021)
Malacostracid (isopod- *Idotea balthica*)	Grazer/scraper	*H. elongata*	MB	NS	Welsh *et al*. (2014)
Maxillopod (barnacle - *Austrominius modestus*)	Filter/suspension	*Renicola roscovita*	MB	Y	Goedknegt *et al*. (2015)
Maxillopod (barnacle - *A. modestus*)	Filter/suspension	*Echinostephilla patellae*	MB	Y	Prinz *et al*. (2009)
Maxillopod (barnacle - *A. modestus*)	Filter/suspension	*P. acanthus*	MB	Y	Prinz *et al*. (2009)
Maxillopod (barnacle - *Semibalanus balanoides*)	Filter/suspension	*H. elongata*	MB	NS, NS	Welsh *et al*. (2014, 2017)
Molluscs					
Clam (*Sphaerium* sp.)	Filter/suspension	*Apatemon sp*.	FW	NS	Selbach *et al*. (2019)
Clam (*Sphaerium* sp.)	Filter/suspension	*Telogaster opisthorchis*	FW	NS	Selbach *et al*. (2019)
Clam (*Sphaerium* sp.)	Filter/suspension	*Aporocotylid sp. II*	FW	NS	Selbach *et al*. (2019)
Clam (*Austrovenus stutchburyi*)	Filter/suspension	*Philophthalmus sp*.	MB	NS	Vielma *et al*. (2019)
Soft-shelled clam (*Mya arenaria*)	Filter/suspension	*H. elongata*	MB	Y	Thieltges *et al*. (2008a)
Common cockle (*Cerastoderma edule*)	Filter/suspension	*H. elongata*	MB	NS	Welsh *et al*. (2019)
	Filter/suspension	*H. elongata*	MB	NS, NS, Y, Y	
Pacific oyster (*Crassostrea gigas*, also known as *Magallana gigas*)					Welsh *et al*. (2014, 2017) and Thieltges *et al*. (2008a, 2009)
Pacific oyster (*Crassostrea gigas*, also known as *Magallana gigas*)	Filter/suspension	*R. roscovita*	MB	Y	Goedknegt *et al*. (2015)
Mussels (*Anodonta anatina*)	Filter/suspension	*D. pseudospathaceum*	FW	Y	Gopko *et al*. (2017)
Mussels (*Dreissena polymorpha*)	Filter/suspension	*Trichobilharzia* sp.	FW	NS	Stanicka *et al*. (2021)
American slipper limpet (*Crepidula fornicata*)	Filter/suspension	*H. elongata*	MB	Y, Y	Thieltges *et al*. (2008a, 2009)
Snail (*Physa acuta*)	Grazer/scraper	*Coitocaecum parvum*	FW	NS	Selbach *et al*. (2019)
Snail (*P. acuta*)	Grazer/scraper	*Trichobilharzia sp*.	FW	NS	Stanicka *et al*. (2021)
Cnidaria					
*Hydra* sp.	Predator	*R. ondatrae*	FW	NS	Schotthoefer *et al*. (2007)
Sea anemone (*Anthopleura aureoradiata*)	Predator	*Curtuteria australis*	MB	Y	Mouritsen & Poulin (2003)
Sea anemone (*A. aureoradiata*)	Predator	*Philophthalmus* sp.	MB	NS	Vielma *et al*. (2019)
Sea anemone (*A. aureoradiata*)	Predator	*Maritrema novaezealandensis*	MB	Y, N	Hopper *et al*. (2008) and Studer *et al*. (2013)
Sea anemone (*Actinia equina*)	Predator	*Echinostephilla patellae*	MB	Y	Prinz *et al*. (2009)
Sea anemone (*A. equina*)	Predator	*P. acanthus*	MB	Y	Prinz *et al*. (2009)
Other invertebrates					
Oligochaete worm (*Chaetogaster limnaei*)	Predator	*Fasciola hepatica*	FW	NS	Rajasekariah (1978)
Oligochaete worm (*C. limnaei*)	Predator	*S. mansoni*	FW	NS	Michelson (1964)
Oligochaete worm (*C. limnaei*)	Predator	*E. trivolvis*	FW	Y	Hopkins *et al*. (2013)
Oligochaete worm (*C. limnaei*)	Predator	*Notocotylid cercariae*	FW	NS	McKoy *et al*. (2011)
Oligochaete worm (*C. limnaei*)	Predator	*Halipegus occidualis*	FW	NS	Fernandez *et al*. (1991)
Turbellarian flatworm (*Macrostomum gigas*)	Scavenger/opportunist	*S. mansoni*	FW	NS	Holliman & Mecham (1971)
Rotifer (*Asplanchna* sp.)	Filter/suspension	*D. pseudospathaceum*	FW	NS	Mironova *et al*. (2019)
Rotifer (*Eosphora ehrenbergi*)	Filter/suspension	*Plagiorchis sp*.	FW	NS	Tokobaev *et al*. (1979)
Bryozoan (*Conopeum tenuissimum*)	Filter/suspension	*Notocotylid cercariae*	FW	NS	Stoddart (1982)
Bryozoan (*C. tenuissimum*)	Filter/suspension	*Strigeid cercariae*	FW	NS	Stoddart (1982)

Habitat type: FW, freshwater; MB, marine/brackish. Transmission effect: Y, yes; N, no; NS, not studied.

## Box 1: Approaches for studying the consumption of cercariae.

Studies of cercariae consumption have been overwhelmingly laboratory-based as these settings typically offer greater control and are well-suited for questions related to precision/quantification. However, some have considered the gut contents of wild-caught consumers (e.g. Kaplan *et al*., 2009). Experimental arenas used in empirical studies have ranged from relatively small sizes, such as individual wells of tissue culture plates (e.g. Hopper *et al*., 2008) and 60–100 ml containers (e.g. Orlofske *et al*., 2012), to large sizes such as 5–6 l containers (e.g. Prinz *et al*., 2009). This is an important consideration as arena size likely has strong effects on the probability of encounter between cercariae and consumers, especially for behavioural influences. For instance, very small volumes are unlikely to account for microhabitat preferences by either party, favouring high contact.

Some studies allowed consumers to acclimate from 4 h (e.g. Mironova *et al*., 2019) to 24 h (e.g. Goedknegt *et al*., 2015) before feeding. Similarly, potential consumers/predators are often, but not always, starved for 24 h (e.g. Kaplan *et al*., 2009; Orlofske *et al*., 2012) or 48 h (e.g., Selbach *et al*., 2019) beforehand. Feeding trial length also affects encounter probability and has been as short as 15–30 min (e.g. Kaplan *et al*., 2009; Orlofske *et al*., 2012) and as long as 72 h (Prinz *et al*., 2009). Studies reaching the limits of cercariae lifespan may reflect a shift from removal by predators to non-active consumption, or even cercariae degradation that opens up their ingestion by other organisms.

Cercariae-related experimental considerations largely pertain to their density and age. Recently-emerged cercariae (<2 h, e.g. Selbach *et al*., 2019) are usually used so they are still relatively active and can attract predator attention. Most studies to date have used only single cercariae densities in feeding trials. This can be as few as two cercariae in a 4-ml well (Selbach *et al*., 2019) to ~6000 cercariae/l (Gopko *et al*., 2017). However, some studies have employed multiple densities for the same cercariae (e.g. Born-Torrijos *et al*., 2020). Cercariae density manipulation studies have overwhelmingly kept constant the volume of water used. An alternative is to keep cercariae numbers constant, manipulating their density by using different volumes of water, but this has not been explored. Given that these approaches can result in different transmission dynamics to downstream hosts (Karvonen *et al*., 2003) and the fact that cercariae consumption ultimately affects the total numbers of cercariae in a given system, this may warrant future investigations. Cercariae density also affects their ability to aggregate, which can in turn influence consumption rate (Orlofske *et al*., 2015).

Measurements of cercariae consumption generally involve either counts of remaining cercariae, or detecting significant reductions in transmission to downstream hosts. The latter usually relies on counting cysts (metacercariae) in/on second intermediate hosts. While directly quantifying transmission, this approach does not measure the pure effect of cercariae consumption because it also integrates potential effects of cercariae consumers on the hosts. For example, predators may not only consume cercariae, but also affect the behaviour of the downstream host, leading to different transmission dynamics. Some feeding experiment studies verified their ability to recover all cercariae in the absence of predators (e.g. Orlofske *et al*., 2012). This is essential in order to conclude that any ‘absent’ cercariae were truly removed rather than missed during enumeration. Many studies visually count cercariae under a dissecting microscope, sometimes using zooplankton counting chambers (e.g. Gopko *et al*., 2017). Filtering large volumes of water to capture cercariae may be helpful, as well as staining (e.g. by rose bengal) to increase their visibility while counting (e.g. Welsh *et al*., 2014).

If reduced transmission is the metric of interest, focal hosts are dissected and examined, but radioisotope labelling of cercariae has also been used (e.g. Christensen, 1979). Cercariae labelling may prove highly useful for detecting cryptic consumption, particularly in complex experimental settings with multiple species and/or large volumes. For instance, Schultz & Koprivnikar (2021) added Carbon-13-labelled cercariae as a potential food source to experimental mesocosms containing a model freshwater food web.
